# Prediction models established for net energy and standardized ileal digestible amino acids in regionally sourced fermented soybean meal for growing pigs

**DOI:** 10.5713/ab.25.0485

**Published:** 2025-09-30

**Authors:** Wenjun Gao, Qile Hu, Yingying Li, Hongrui Cao, Xue Bao, Renjie Wang, Shuai Zhang

**Affiliations:** 1 State Key Laboratory of Animal Nutrition and Feeding, Ministry of Agriculture and Rural Affairs Feed Industry Centre, China Agriculture University, Beijing, China; 2 DadHank (Chengdu) Biotech Corp., Chengdu, China; 3 Frontiers Science Center for Molecular Design Breeding (MOE), China Agricultural University, Beijing, China

**Keywords:** Amino Acids, Available Energy, Fermented Soybean Meal, Growing Pigs

## Abstract

**Objective:**

The study was conducted to determine the available energy and standardized ileal digestibility (SID) of amino acid (AA) in fermented soybean meal (FSBM), and establish prediction equations for growing pigs.

**Methods:**

In Exp. 1, to determine available energy, twenty-four growing barrows (initial body weight = 35.3±3.2 kg) were randomly assigned to two replicated 6×6 Latin square designs, each comprising one corn-based basal diet (used in both squares) and five test diets. In Exp. 2, on AA digestibility, twenty-two growing barrows (initial body weight: 48.8± 2.8 kg) underwent distal ileal T-cannulation and were arranged to a 3-period crossover design with one nitrogen-free diet and 10 test diets. The inclusion levels of FSBM in the test diets of Exp. 1 and Exp. 2 were 27.49% and 40%, respectively.

**Results:**

The coefficients of variation among FSBM for ether extract (EE), crude fiber (CF), neutral detergent fiber (NDF), and acid detergent fiber (ADF) all exceeded 10%. The digestible energy, metabolizable energy, and net energy (NE) values of FSBM ranged from 15.50 to 18.44 MJ/kg dry matter (DM), 13.98 to 16.72 MJ/kg DM, and 10.10 to 11.05 MJ/kg DM, respectively. The SID values of AA demonstrated variation (p<0.05) for most AA, with the exception of Arg and Lys among indispensable AA, and Glu, Ser, and Tyr among dispensable AA. The best-fitted prediction equation for NE was a model incorporating EE, NDF, and gross energy (R^2^ = 0.92, p<0.01), while the best-fitted equations for SID_Crude Protein_ and SID_Lys_ incorporated DM, NDF, and ADF (R^2^ = 0.71, p = 0.097) and CF, ADF, and Ash (R^2^ = 0.83, p = 0.022), respectively.

**Conclusion:**

The NE values of FSBM ranged from 10.10 to 11.05 MJ/kg DM. The NE value, SID_Crude Protein_, and SID_Lys_ of FSBM can be well predicted based on nutritional parameters.

## INTRODUCTION

Fermented soybean meal (FSBM), a high-quality protein ingredient, is produced through microbial fermentation of soybean meal (SBM). This bioprocess effectively degrades anti-nutritional factors (ANF) and produces beneficial components such as small peptides and probiotic bacteria [1]. Given the global shortage of SBM and the increasing demand for alternative protein feed resources, FSBM has attracted considerable research interest due to its superior digestibility and cost-effectiveness.

Extensive research suggests that FSBM provides multiple benefits for weaned piglets, including improved growth performance [2–4], reduced diarrhea incidence [2,5], enhanced intestinal health [6,7], improved intestinal morphology [8], and reduced mortality rates [9,10]. However, although these benefits for weaned piglets are established, key information is still lacking on the value of FSBM for growing pigs, especially concerning its net energy (NE) value and the standardized ileal digestibility (SID) values of amino acid (AA), which are further complicated by heterogeneity of fermentation processes and testing standards.

Compared with the digestible energy (DE) and metabolizable energy (ME) systems, the NE system more accurately reflects the true energy value of ingredients [11–13]. Therefore, determining the NE value of FSBM would better demonstrate its economic value. Although prior studies have focused on the nutrient composition of FSBM, using stepwise regression to predict its NE from this composition remains unexplored. For AA digestibility, the SID, which accounts for basal endogenous losses (BEL), is more accurate than apparent ileal digestibility (AID) and is widely used in research [14,15]. However, investigations into the SID of FSBM in swine nutrition remain limited.

Hence, this study aimed to establish a database for FSBM and develop prediction equations for estimating these nutritional parameters based on its chemical composition. Specifically, we will carry out the following tasks: (1) evaluate DE, ME, and NE values, (2) evaluate AID and SID of AA, and (3) develop prediction equations of FSBM samples.

## MATERIALS AND METHODS

### Collection of fermented soybean meal samples

Ten FSBM samples were collected from the major produced provinces across China, representing the following regions: Guangdong (sample 1), Sichuan (samples 2 and 10), Hubei (sample 3), Jiangsu (samples 4 and 5), Shandong (samples 6 and 7), Hebei (sample 8), and Tianjin (sample 9). All FSBM samples were prepared by the strain fermentation method and maintained under dry conditions. Owing to commercial confidentiality, detailed microbiological data for the FSBM samples could not be obtained. A total of 10 samples were initially collected and utilized in feeding trials, however, subsequent analysis identified that sample 10 was a distinct protein ingredient. This sample was consequently excluded, leaving 9 FSBM samples for the final analysis (Table 1).

### Exp. 1: Available energy experiment

*Experimental animals and diets:* Twenty-four growing barrows (Duroc×Landrace×Yorkshire) with an initial body weight (BW) of 35.3±3.2 kg were used in this experiment. The experimental diets included a corn-based basal diet and ten test diets formulated by replacing 27.49% of the corn with different FSBM samples. The available energy value of FSBM was calculated using the difference method while maintaining constant levels of dicalcium phosphate, limestone, salt, and the vitamin-mineral premix (Table 2).

*Experimental design and procedure:* In this experiment, twenty-four pigs were randomly assigned to two replicated 6×6 Latin square design, with 12 pigs in each square. The pigs in each square were divided into two groups of six, and the two groups entered six similar open-circuit respiration chambers in a crossover manner over six periods. This design provided six replicates per treatment within each square. Each square consisted of six experimental diets: one corn-based basal diet (common to both squares) and five test diets containing different FSBM samples. Thus, while the two Latin squares collectively involved a total of 12 diets, the shared use of the same basal diet across both squares resulted in a total of 11 experimental diets. Throughout each period, all six diets within a square were simultaneously evaluated, with each diet randomly assigned to one respiration chamber.

Each period consisted of 10 days: 5 days for dietary adaptation, 1 day for chambers adaptation, 3 days for feces/urine collection and gas measurement (recording daily O_2_ consumption along with CO_2_ and CH_4_ production), followed by the last day when fasting heat production (FHP) was determined during the last 8 hours from 2230 (day 9) to 0630 (day 10). Pigs were fed one of five experimental diets formulated at 4% of BW, provided as equal-sized meals twice daily at 0830 and 1530, with feed amounts adjusted based on BW measured on days 0, 5, and 10.

*Sample collection*: During the collection period (days 7 to 9), residual and spilled feed were collected, dried, and weighed daily. Feces were collected each morning at 0830 when the chamber door was opened and immediately stored at −20°C. Urine was collected concurrently into plastic buckets containing 50 mL of *6 N* HCl. Then, it was filtered through cotton gauze, and the total volume was recorded while a 5% aliquot was preserved at −20°C. For FHP determination (days 9 to 10), additional 24 h fasting-state urine collections were conducted. At the end of the collection period, all samples were thawed and homogenized. All measurements were carried out according to the methods described by Zhang et al [16] and Noblet [17].

*Chemical analyses:* The FSBM samples, basal diet, and 10 test diets used in Exp. 1 were analyzed for dry matter (DM, procedure 930.15; AOAC International [18]), crude protein (CP = nitrogen×6.25, procedure 984.13; AOAC International [18]), ether extract (EE; Lyu et al [19]) and ash (procedure 923.03; AOAC International [18]). The neutral detergent fiber (NDF) and acid detergent fiber (ADF) were determined using a fiber analyzer (ANKOM Technology) according to a modification of the procedures of Van Soest et al [20]. The gross energy (GE) of all samples were determined using an adiabatic oxygen bomb calorimeter (Parr Instruments).

*Calculations:* The apparent total tract digestibility (ATTD) of the experimental diets was calculated according to the methods of Noblet et al [21].


(1) $$\text{ATTD}=([{\text{F}}_{\text{i}}-{\text{F}}_{\text{f}}]/{\text{F}}_{\text{i}})\times 100\%$$

where F_i_ and F_f_ are the total intake and fecal output of energy (MJ) or nutrients (gram), respectively, during the collection period.

The DM intake (DMI) from d 7 to 9 in each period was determined by multiplying feed intake by the DM content of diets. The DE of each experimental diet was calculated by subtracting fecal energy from GE. The ME was derived by subtracting urinary energy (UE) and methane energy (CH_4_E) from DE. The NE values of each experimental diet were calculated based on gas data: concentrations of O_2_, CO_2_, and CH_4_ were averaged separately for the fed period and the last 8 h of fasting. Respiratory quotient was derived as the ratio of CO_2_ production to O_2_ consumption (L/d) [22]. Total heat production (THP) and FHP were calculated using established equations [22]. The RE as protein (RE_P_) was calculated from nitrogen retention (nitrogen×6.25×5.70, kcal/g), whereas the amount of the RE as lipids (RE_L_) was calculated as the difference between RE and RE_P_. All methodologies followed Li et al [11], Lyu et al [13], and Adeola [23]. The main calculations are presented as follows:


(2) $$\text{THP}/\text{FHP}=3.87\times {\text{O}}_{2}+1.20\times {\text{CO}}_{2}-0.53\times {\text{CH}}_{4}-1.43\times \text{Urinary nitrogen excretion}$$

(3) NE=(ME-THP+FHP)/DMI

The difference method was used to calculate the available energy values and ATTD contributions of FSBM, assuming that the minerals and vitamins in the experimental diets were sources of nutrients but not sources of energy [23].


(4) $$\text{Energy }{\text{value}}_{\text{FSBM}}=(\text{Energy }{\text{value}}_{\text{test diet}}-\text{Energy }{\text{value}}_{\text{basal diet}}/{\text{r}}_{\text{0}}\times {\text{r}}_{1})/{\text{r}}_{2}$$

(5) $${\text{ATTD}}_{\text{FSBM}}=(\{{\text{ATTD}}_{\text{test diet}}-[100-{\text{r}}_{2}]\times {\text{ATTD}}_{\text{basal diet}}\})/{\text{r}}_{2}$$

where the energy value_test diet_ and energy value_basal diet_ are the available energy values of the test and basal diets, respectively; r_0_ = the corn fraction in the basal diet, r_1_ = the corn proportion in the test diet, and r_2_ = the FSBM proportion in the test diet. The ATTD_test diet_ and ATTD_basal diet_ values are the ATTD of nutrients in the test and basal diets, respectively.

*Statistical analyses:* All data for the experiment were analyzed using analysis of variance with the Proc MIXED of SAS (SAS Institute). The LSMEANS statement with Tukey’s adjustment was used to separate mean values. In all analyses, the differences were considered significant if p<0.05. The relationship between NE and chemical composition of FSBM samples was determined using Proc CORR of SAS. The prediction equations for NE of FSBM samples were developed using Proc REG of SAS. The coefficient of determination (R^2^), root mean square error (RMSE) and Akaike’s information criterion (AIC) were used as the selection criterion for the best fit equations.

### Exp. 2: Amino acid digestibility experiment

*Animals, diets and experimental design:* In this experiment, twenty-two growing barrows (Duroc×Landrace×Yorkshire; initial BW = 48.8±2.8 kg), each fitted with a T-cannula near the distal ileum, were used in a 3-period crossover design to evaluate 11 experimental diets. The experimental diets included one nitrogen-free diet and 10 FSBM test diets. The test diets contained 40% of one of the nine FSBMs as the sole source of protein, while the nitrogen-free diet was formulated according to the methods described by Stein et al [24] to estimate BEL for subsequent determination of SID for CP and AA (Table 3). The surgical implantation of T-cannulas near the distal ileum was carried out following the established procedures described by Stein et al [25]. Each period lasted 7 days. During this period, there were 5 days for dietary adaptation, followed by 2 days for ileal digesta collection from 0800 to 1700. All collection procedures adhered to the description provided by Stein et al [25].

*Sample preparation and chemical analyses:* Chromic oxide (Cr_2_O_3_, 0.3%) was added as an indigestible marker for AA digestibility calculations. Diet and ileal digesta samples from Exp. 2 were hydrolyzed with *6 N* HCl (110°C, 24 h) and analyzed for 15 AA using an Amino Acid Analyzer (Hitachi L-8900) [18]. For the sulfur-containing AA, Met and Cys were pre-oxidation with performic acid prior to hydrolysis with 7.5 *N* HCl under the same conditions, and then analyzed using a Hitachi L-8800 analyzer. Moreover, Trp was determined by High Performance Liquid Chromatography (Agilent 1200 Series; Agilent Technologies) after LiOH hydrolysis (110°C, 22 h). The Cr concentration in samples was determined by Polarized Zeeman Atomic Absorption Spectrometer (Hitachi Z2000) following nitric–perchloric acid wet-ashing.

*Calculations:* The AID of AA in the diets containing FSBM was calculated according to the following equation [24]:


(6) $$\text{AID}=(1-[{\text{AA}}_{\text{digesta}}/{\text{AA}}_{\text{diet}}]\times [{\text{Cr}}_{\text{diet}}/{\text{CR}}_{\text{digesta}}])\times 100\%$$

where AID = the apparent ileal digestibility of an AA or CP (%), AA_digesta_ = the AA concentration in the ileal digesta (g/kg of DM), AA_diet_ = the AA concentration in the diets (g/kg of DM), Cr_diet_ = the chromium concentration in the diet (g/kg of DM), and Cr_digesta_ = the chromium concentration in the ileal digesta (g/kg of DM).


(7) $$\text{BEL}={\text{AA}}_{\text{digesta}}\times ({\text{Cr}}_{\text{diet}}/{\text{Cr}}_{\text{digest}})$$

(8) $$\text{SID}=\text{AID}+(\text{BEL}/{\text{AA}}_{\text{diet}})\times 100\%$$

where BEL = the basal endogenous losses of an AA or CP (g/kg of DM intake), and SID = the standardized ileal digestibility of an AA (%).

*Statistical analyses:* The statistical analysis for Exp. 2 followed the same methods as Exp. 1. Data for AID and SID were analyzed using Proc MIXED in SAS with Tukey’s test for mean separation. Relationships between digestibility values and chemical composition were assessed using Proc CORR, and prediction equations for SID were developed using Proc REG, with R^2^, RMSE, and AIC as model selection criteria.

## RESULTS

### Chemical analysis

The chemical composition of FSBM varied among samples, with the coefficient of variation (CV) greater than 10 % for EE, crude fiber (CF), NDF and ADF (Table 1). The CP content of the nine FSBM samples averaged 49.82%, and ranged from 48.02% to 51.10%. The CV values of the 18 types of AA were all below 10%. The average values of Lys and Met were 2.84% and 0.74%, respectively.

### Exp. 1: Available energy experiment

*Nutrients digestibility and nitrogen balance for diets:* The ATTD of DM and organic matter (OM) in FSBM 4 diet was higher than that of FSBM 8 diet (p<0.05), whereas both FSBM 4 and FSBM 9 diets exhibited greater ATTD of GE than the FSBM 8 diet (Table 4). For ATTD of CP, all FSBM diets except FSBM 8 diet were superior to the basal diet (p<0.05). The addition of FSBM increased nitrogen intake and nitrogen retention compared to the basal diet (p<0.05). Although fecal output was highest in FSBM 8 diet (10.57 g/d), FSBM 1, 6 and 8 diets showed higher fecal output than the basal diet (p<0.05).

*Energy balance and energy value for experimental diets:* The ME intake and FHP were not affected by dietary treatments (Table 5). Pigs fed FSBM 1, 4, 6, and 9 diets showed higher THP than those fed the basal diet, whereas the FSBM 8 diet resulted in lower THP than FSBM 4 diet (p<0.05). For RE_P_, all FSBM diets showed higher values compared to the basal diet (p<0.05). The NE-to-ME ratio in FSBM 3 diet, FSBM 4 diet, and FSBM 5 diet was lower than that of basal diet (p<0.05). However, no differences were observed in the ME-to-DE ratio and UE-to-DE ratio.

*Nutrient digestibility and energy contents for ingredients:* No differences were observed in the energy utilization among the FSBM samples (Table 6). Consistent with the dietary results, the ATTD of DM and OM was higher in FSBM 4 than in FSBM 8 (p<0.05). With the exception of FSBM 6, all other FSBM samples exhibited a higher ATTD of CP compared to FSBM 8 (p<0.05). Furthermore, the ATTD of ADF was higher in FSBM 3, 4, 5, and 9 than in FSBM 8 (p<0.05). The FSBM 4 and 9 showed higher DE value than that of FSBM 1, 6, and 8 (p<0.05). The DE, ME, and NE values of FSBM samples ranged from 15.50 to 18.44 MJ/kg DM, 13.98 to 16.72 MJ/kg DM, and 10.10 to 11.05 MJ/kg DM, respectively. The average NE value of the FSBM ingredient was 10.65 MJ/kg DM.

### Exp. 2: Amino acid digestibility experiment

*Apparent ileal digestibility and basal endogenous losses in growing pigs:* There were differences in the AID values of Lys, Met, and Trp among the indispensable AA, as well as in those of Asp and Tyr among the dispensable AA (Table 7). The AID of Trp and Asp was higher in FSBM 9 than that of FSBM 1 and 6 (p<0.05). The AID of Tyr in FSBM 9 was higher than in FSBM 6 (p<0.05). The BEL values for indispensable AA ranged from 0.16 g/kg DMI for Trp to 0.79 g/kg DMI for Leu. For dispensable AA, the BEL ranged from 0.05 g/kg DMI for both Ser and Tyr to 0.27 g/kg DMI for Glu.

*Standardized ileal digestibility:* Statistical analysis revealed that the SID values of AA demonstrated variation among FSBM samples (p<0.05) for most AA, with the exception of Arg and Lys among indispensable AA, and Glu, Ser, and Tyr among dispensable AA (Table 8). The SID of Met was highest in FSBM 9, surpassing that of FSBM 3, 4, 5, 6, and 8 (p<0.05). Similarly, the SID values for Iso, Phe, and Trp in FSBM 9 were also greater compared to FSBM 1 and 6 (p<0.05).

*Correlation analysis and prediction equations for standardized ileal digestible amino acids and net energy in fermented soybean meal samples:* In terms of energy values, the DM content exhibited positive correlations with both GE (r = 0.75, p< 0.05) and NE (r = 0.78, p<0.05; Table 9). Regarding the SID of AA, the ash showed a negative correlation with SID_Lys_ (r = −0.70, p<0.05), whereas the NDF had a positive correlation with SID_Lys_ (r = 0.69, p<0.05).

The regression equations for NE in the current experiment were developed based on chemical composition and GE (Table 10). Three robust models emerged: (1) an NDF-GE model (R^2^ = 0.81, p<0.01), (2) an EE-GE model (R^2^ = 0.85, p<0.01), and (3) a comprehensive model incorporating EE, NDF and GE (R^2^ = 0.92, p<0.01). The best-fit predictive equations for SID_CP_ and SID_Lys_ were as follows: SID_CP_ = 117.42–0.58×DM–0.40×NDF+2.99×ADF (R^2^ = 0.71, p = 0.079) and SID_Lys_ = 196.63+1.56×CF+6.08×ADF–24.56×Ash (R^2^ = 0.83, p = 0.022), respectively.

## DISCUSSION

### Chemical composition of ingredient

The fermentation process enhances the nutritional and functional qualities of SBM. It not only eliminates ANF, reducing their negative impact on digestion, but also enables microbial proteases to break down soybean protein into highly absorbable peptides and free AA, greatly improving digestibility and utilization [2].

The nutrient composition (such as EE, CF, NDF, and ADF) of the FSBM samples exhibited notable variability, likely due to the differences in the sources or strains of SBM used in the fermentation process [26]. Variability in fiber content may stem from inherent differences in soybean composition, inconsistent removal of seed coats, and varying microbial degradation efficiency during fermentation. The average EE content observed was lower than that reported by NRC [27] (1.16% vs. 2.30%), possibly reflecting improvements in modern oil extraction technology. The Lys concentration was consistent with values reported by Yan et al [28] and Huang et al [15], but lower than that reported by Jang and Kim [29]. Variations in Lys content across studies may be attributed to differences in fermentation processes, microbial strains, and analytical methods.

### Nutrient digestibility and nitrogen balance for experimental diets and ingredients

The lower ATTD of DM, OM, GE, and CP observed in the FSBM 8 diet, compared to the FSBM 4 diet, can be attributed to differences in nutrient compositions. The higher fiber content (CF, NDF, and ADF) likely physically hindered enzymatic access to nutrients, thereby reducing overall digestibility. Consistent with this finding, the ATTD values of the FSBM samples calculated using the difference method showed similar trends: the FSBM 8 sample exhibited lower ATTD of GE, CP, ADF, and OM than the FSBM 4 sample.

Compared to the basal diet, all FSBM groups showed higher nitrogen intake (42.3 vs. 15.2 g/d) and retention (21.0 vs. 4.2 g/d), consistent with the positive correlation between CP levels and nitrogen utilization. Fecal nitrogen output primarily consists of undigested dietary nitrogen, endogenous losses, and microbial protein [30]. The FSBM 8 diet resulted in higher fecal nitrogen output (10.6 vs. 6.4 g/d on average) and lower ATTD of CP (78.0% vs. 86.7% on average) than other FSBM diets. This may be due to: 1) excessive fermentation-induced protein denaturation and Maillard reactions, and 2) specific microbial strains that increased indigestible microbial protein. These results highlight that the nutritional value of FSBM depends not only on CP level, but also on the overall nutrient composition and the effectiveness of the fermentation process.

### Energy balance and energy value for experimental diets and ingredients

The RE_P_ values were consistent with nitrogen retention, indicating that the addition of FSBM increased RE_P_. This improvement can be attributed to the enhanced AA balance in the diet, which effectively promotes protein deposition.

The available energy of FSBM samples was calculated using the difference method, which assumes no interaction between the basal diet and test ingredient. However, in reality, such interactions, particularly from CP, influence energy values. Studies indicate that higher dietary CP levels increase whole-body protein turnover and urinary nitrogen excretion, leading to greater heat increment [21]. Therefore, when applying the difference method to determine the energy value of protein-rich ingredients like FSBM, the difference in CP content between the basal diet and test diets is critical for accurately estimating energy value [30].

The average ME value of the FSBM samples (15.63 MJ/kg DM) was lower than the value listed in Nutrient Requirements of Swine in China (16.52 MJ/kg DM) [31]. This discrepancy can be attributed to two main factors: the lower EE content in the FSBM samples used in this study (1.27% vs. 2.30%), and the higher CP level (average 19.77%) in the FSBM diets. As reported by Kim et al [32], when dietary CP exceeds 17.0% in growing pigs, UE excretion increases, which may result in an underestimation of ME and possibly NE of the test ingredient. Among the different FSBM samples, the DE values of FSBM 8 were lower than those of FSBM 4 and 9. This difference may be attributed to the higher fecal output observed in FSBM 8, as fecal output is negatively correlated with DE [17].

### Amino acid digestibility

The comprehensive analysis of AID, BEL, and SID in the present study offers valuable insights into the AA availability of the FSBM samples evaluated. Samples such as FSBM 9 exhibited higher digestibility values than FSBM 1 and 6 in Trp and Asp, suggesting that differences in fermentation conditions, possibly related to microbial strains, process parameters, or substrate composition, distinctly influenced the breakdown of protein fractions and ANF.

The comprehensive analysis of AID, BEL, and SID in the present study offers valuable insights into the AA availability of the FSBM samples evaluated. Significant variations were observed among samples; for instance, the AID of Lys was higher in FSBM 2, 4, and 9 than in FSBM 1 (p<0.05), and the AID of Tyr in FSBM 9 was higher than in FSBM 6 (p<0.05). Samples such as FSBM 9 consistently exhibited superior digestibility values, suggesting that differences in fermentation conditions, possibly related to microbial strains, process parameters, or substrate composition, distinctly influenced the breakdown of protein fractions and ANF.

The BEL values obtained in this study were generally lower than those reported in other studies with growing pigs. For instance, the mean BEL of CP determined in the present study was 5.20 g/kg DMI, substantially lower than the values of 10.53 g/kg DMI reported by Jansman et al [33] and 17.10 g/kg DMI reported by Park et al [34]. Similar trends were observed for many AA, with the mean BEL of Val (0.26 vs. 0.41 g/kg DMI), Thr (0.19 vs. 0.51 g/kg DMI), and Pro (0.08 vs. 1.31 g/kg DMI) being lower than those reported by Jansman et al [33]. It is noteworthy that despite these lower mean values, all BEL values for AA obtained in this study fell within the ranges reported in these meta-analyses [33,34]. Given that all these results were based on the nitrogen-free diet, the considerable variations could be attributed to other factors such as animal age, initial BW, dietary composition, and feeding level [29,34,35]. These variations in BEL are critical to acknowledge, as lower BEL values may amplify the impact of incomplete digestion on SID estimates.

After correcting for BEL, the SID values provided a more accurate estimation of the true digestibility of AA from the FSBM samples. The SID of Met (77.5%) in this study was lower than that of NRC [27] (77.5% vs. 88%) and Nutrient Requirement of Swine in China [31] (77.5% vs. 91%), however, consistent with the research (77.5% vs. 75.3%) by Huang et al [15]. Meanwhile, the SID of Lys (74.7%) in this study remains well within the published range for FSBM, with documented values for SID of Lys ranging from 75% to 88% [29] to approximately 77% [29], which further confirms the plausibility of our results. However, existing studies show certain variations in the SID of AA, which may be attributed to differences in processing and fermentation conditions, methodological influences, and matrix effects [36].

### Correlations and prediction equations

Our results demonstrated a negative relationship between EE and NE, which may be attributed to the low content of EE in the FSBM samples, limiting its contribution to NE and thus its role in energy prediction. More notably, a positive correlation was observed between NDF and the SID_Lys_ (r = 0.69, p<0.05), while the relationship between ADF and SID_Lys_ also showed a positive, though not statistically significant, trend (r = 0.59, p = 0.097). This finding reflects an important characteristic of the fermentation process: higher fiber content (both NDF and ADF) may indicate more extensive fermentation, during which soluble nutrients are metabolized by microorganisms, leading to a relative increase in fiber concentration. Therefore, the positive association with SID_Lys_ does not imply that ADF directly enhances digestibility; rather, it serves as an indirect marker of stronger microbial activity.

Although this study has established prediction equations for NE, SID_CP_, and SID_Lys_ in FSBM for growing pigs, the relatively low R^2^ values of the models for SID_CP_ and SID_Lys_ indicate limited explanatory power. Thus, further research incorporating larger and more diverse datasets is necessary to improve the accuracy and robustness of these predictive equations.

Several limitations of this study should be acknowledged. The absence of data on critical ANF limits interpretation of the variability in digestibility across FSBM samples. Additionally, commercial confidentiality surrounding microbial strains and fermentation parameters hindered further investigation into the causes of product differences. Future research should combine detailed ANF analysis with standardized digestibility assays and promote greater transparency in fermentation protocols to improve reproducibility and enable reliable cross-study comparisons.

## CONCLUSION

In summary, the chemical composition of FSBM varied across samples, particularly with regard to the concentrations of EE, CF, NDF, and ADF. The SID values of AA demonstrated variation among FSBM samples for most AA. Furthermore, the NE values of FSBM ranged from 10.10 to 11.05 MJ/kg DM. The NE value, SID_CP_ and SID_Lys_ of FSBM could be predicted based on their nutritional parameters.

## Figures and Tables

**Table 1:** Analyzed nutrient composition and source of fermented soybean meal (FSBM) (as-fed basis)

Items	FSBM1	FSBM2	FSBM3	FSBM4	FSBM5	FSBM6	FSBM7	FSBM8	FSBM9	Mean	CV
Source	Guangdong	Sichuan	Hubei	Jiangsu	Jiangsu	Shandong	Shandong	Hebei	Tianjin		
Nutrient components (%)											
Dry matter	92.0	90.6	88.8	90.1	89.5	92.2	90.1	93.3	93.8	91.2	1.9
Crude protein	49.8	48.8	50.5	50.5	49.6	48.0	51.1	49.6	50.5	49.8	2.0
Ether extract	1.0	1.4	1.8	1.2	0.7	1.3	1.0	0.7	1.3	1.2	30.0
Crude fiber	3.8	4.1	4.6	4.0	3.7	6.2	4.2	5.1	4.3	4.5	17.8
Neutral detergent fiber	7.9	10.9	13.5	8.6	8.0	11.8	12.8	11.6	12.5	10.8	19.9
Acid detergent fiber	4.4	5.2	5.7	4.7	4.5	4.5	5.6	5.7	5.8	5.1	11.7
Ash	6.8	6.3	6.5	6.4	6.7	6.5	6.4	6.8	6.7	6.6	2.8
Gross energy (MJ/kg)	18.5	18.3	18.2	18.3	18.0	18.6	18.5	18.4	19.1	18.4	1.7
Indispensable amino acids (%)											
Arginine	3.4	3.3	3.4	3.3	3.4	3.2	3.2	3.0	2.7	3.2	6.8
Histidine	1.3	1.2	1.3	1.3	1.3	1.2	1.2	1.1	1.1	1.2	6.5
Isoleucine	2.3	2.3	2.4	2.4	2.3	2.2	2.3	2.1	2.0	2.3	6.1
Leucine	3.8	3.7	3.9	4.0	3.8	3.6	3.8	3.5	3.3	3.7	6.1
Lysine	2.9	2.8	3.0	3.1	3.0	2.8	3.0	2.7	2.3	2.8	8.0
Methionine	0.7	0.8	0.7	0.8	0.8	0.7	0.7	0.7	0.7	0.7	2.7
Phenylalanine	2.5	2.5	2.6	2.7	2.6	2.5	2.6	2.3	2.2	2.5	5.9
Threonine	2.0	2.0	2.0	2.1	2.1	1.9	2.0	1.8	1.7	1.9	6.9
Tryptophan	0.6	0.6	0.6	0.6	0.6	0.6	0.6	0.7	0.6	0.6	3.8
Valine	2.5	2.4	2.5	2.5	2.4	2.2	2.4	2.2	2.1	2.3	6.4
Dispensable amino acids (%)											
Alanine	2.4	2.3	2.3	2.4	2.4	2.1	2.3	2.1	2.0	2.3	7.4
Asparagine	5.7	5.5	5.8	5.9	5.8	5.3	5.7	5.2	4.8	5.5	6.5
Cysteine	0.9	0.9	0.9	1.1	0.9	0.9	0.9	0.8	0.8	0.9	9.5
Glutamic acid	9.4	9.1	9.4	9.5	9.6	8.7	9.1	8.5	7.7	9.0	6.9
Glycine	2.2	2.1	2.2	2.3	2.2	2.0	2.1	1.9	1.8	2.1	7.1
Proline	2.7	2.5	2.6	2.7	2.6	2.4	2.5	2.3	2.2	2.5	7.0
Serine	2.6	2.5	2.6	2.7	2.6	2.4	2.5	2.3	2.1	2.5	6.9
Tyrosine	1.7	1.7	1.7	1.7	1.7	1.6	1.6	1.4	1.4	1.6	9.3
Total amino acids	49.5	48.0	50.1	50.8	50.0	46.0	48.4	44.6	41.4		

CV, coefficient of variation.

**Table 2:** Ingredient composition and analyzed nutrient concentrations of the experimental diets in Exp. 1

Items	Basal diet	FSBM diets No.								

		1	2	3	4	5	6	7	8	9
Ingredient composition (%)										
Corn	96.10	68.61	68.61	68.61	68.61	68.61	68.61	68.61	68.61	68.61
Fermented soybean meal	-	27.49	27.49	27.49	27.49	27.49	27.49	27.49	27.49	27.49
Dicalcium phosphate	1.20	1.20	1.20	1.20	1.20	1.20	1.20	1.20	1.20	1.20
Limestone	1.90	1.90	1.90	1.90	1.90	1.90	1.90	1.90	1.90	1.90
Salt	0.30	0.30	0.30	0.30	0.30	0.30	0.30	0.30	0.30	0.30
Vitamin and mineral premix^1)^	0.50	0.50	0.50	0.50	0.50	0.50	0.50	0.50	0.50	0.50
Analyzed nutrient concentrations (%)										
Dry matter	86.8	87.8	87.7	87.7	87.9	87.8	89.3	89.0	89.0	89.2
Crude protein	8.1	19.4	19.4	20.1	19.7	19.9	19.3	20.1	20.0	20.0
Ether extract	3.1	2.7	2.6	2.9	2.8	2.6	2.7	2.8	2.4	3.0
Crude fiber	3.2	3.7	3.9	4.4	4.2	3.2	4.4	4.8	5.3	4.7
Neutral detergent fiber	8.1	8.7	9.0	9.9	8.6	8.4	9.1	8.5	10.6	9.8
Acid detergent fiber	2.8	3.1	3.8	3.8	3.5	3.6	4.0	3.5	4.1	4.1
Ash	4.4	5.8	6.1	5.9	5.6	5.7	6.4	6.2	6.3	6.1
Gross energy (MJ/kg)	15.7	16.2	16.2	16.2	16.2	16.1	16.1	16.0	15.8	16.6

1) Vitamin and mineral premix provided the following per kilogram of complete diet: vitamin A as retinyl acetate, 5,512 IU, vitamin D_3_ as cholecalciferol, 2,200 IU, vitamin E as _DL_-alpha-tocopheryl acetate, 30 IU, vitamin K_3_ as menadione nicotinamide bisulfite, 2.2 mg, vitamin B_12_, 27.6 μg, riboflavin, 4 mg, pantothenic acid as _DL_-calcium pantothenate, 14 mg, niacin, 30 mg, choline chloride, 400 mg, folacin, 0.7 mg, thiamin as thiamine mononitrate, 1.5 mg, pyridoxine as pyridoxine hydrochloride, 3 mg, biotin, 44 μg, Mn as MnO, 40 mg, Fe as FeSO_4_·H_2_O, 75 mg, Zn as ZnO, 75 mg, Cu as CuSO_4_·5H_2_O, 100 mg, I as KI, 0.3 mg, and Se as Na_2_SeO_3_, 0.3 mg.

FSBM, fermented soybean meal.

**Table 3:** Ingredient composition and analyzed nutrient concentrations of the experimental diets in Exp. 2

Items	Nitrogen-free diet	FSBM diets No.								

		1	2	3	4	5	6	7	8	9
Ingredient composition (%)										
Cornstarch	68.90	34.40	34.40	34.40	34.40	34.40	34.40	34.40	34.40	34.40
Fermented soybean meal	-	40.00	40.00	40.00	40.00	40.00	40.00	40.00	40.00	40.00
Sucrose	20.00	20.00	20.00	20.00	20.00	20.00	20.00	20.00	20.00	20.00
Cellulose acetate	4.00	-	-	-	-	-	-	-	-	-
Soybean oil	3.00	3.00	3.00	3.00	3.00	3.00	3.00	3.00	3.00	3.00
Dicalcium phosphate	1.60	1.00	1.00	1.00	1.00	1.00	1.00	1.00	1.00	1.00
Limestone	1.00	0.50	0.50	0.50	0.50	0.50	0.50	0.50	0.50	0.50
Salt	0.30	0.30	0.30	0.30	0.30	0.30	0.30	0.30	0.30	0.30
Vitamin and mineral premix^1)^	0.50	0.50	0.50	0.50	0.50	0.50	0.50	0.50	0.50	0.50
Potassium carbonate	0.30	-	-	-	-	-	-	-	-	-
Magnesium oxide	0.10	-	-	-	-	-	-	-	-	-
Chromic oxide	0.30	0.30	0.30	0.30	0.30	0.30	0.30	0.30	0.30	0.30
Analyzed nutrient concentrations (%)										
Dry matter	89.3	91.0	90.5	90.3	90.6	90.4	90.8	90.8	90.5	91.4
Crude protein	1.0	20.8	20.7	20.5	20.4	20.0	21.8	20.8	21.0	20.6
Indispensable amino acids (%)										
Arginine	-	1.1	1.2	1.4	1.0	0.9	1.1	1.3	1.3	1.5
Histidine	-	0.4	0.5	0.6	0.4	0.4	0.5	0.5	0.5	0.6
Isoleucine	-	0.8	0.9	1.0	0.8	0.7	0.8	0.9	0.9	1.1
Leucine	-	1.3	1.4	1.7	1.3	1.1	1.3	1.5	1.6	1.8
Lysine	-	1.0	1.0	1.2	1.0	0.9	1.0	1.1	1.2	1.3
Methionine	-	0.2	0.2	0.2	0.2	0.2	0.2	0.3	0.3	0.3
Phenylalanine	-	0.9	1.0	1.2	0.9	0.8	0.9	1.1	1.1	1.3
Threonine	-	0.7	0.8	0.9	0.7	0.6	0.8	0.8	0.8	1.0
Tryptophan	-	0.2	0.2	0.2	0.2	0.2	0.2	0.2	0.2	0.2
Valine	-	0.9	0.9	1.1	0.8	0.7	0.8	1.0	1.0	1.2
Dispensable amino acids (%)										
Alanine	-	0.8	0.9	1.0	0.8	0.7	0.8	0.9	0.9	1.1
Asparagine	-	2.0	2.1	2.5	1.9	1.7	2.0	2.3	2.3	2.7
Cystine	-	0.4	0.4	0.4	0.3	0.3	0.3	0.3	0.4	0.3
Glutamic acid	-	3.3	3.5	4.0	3.0	2.8	3.4	3.7	3.8	4.4
Glycine	-	0.8	0.8	0.9	0.7	0.6	0.8	0.8	0.9	1.0
Proline	-	0.9	1.0	1.1	0.9	0.8	0.9	1.0	1.1	1.2
Serine	-	0.9	0.9	1.1	0.9	0.8	0.9	1.0	1.0	1.2
Tyrosine	-	0.6	0.6	0.7	0.5	0.4	0.5	0.6	0.6	0.6
Total amino acids		17.1	18.5	21.1	16.0	14.5	17.2	19.2	19.7	22.7

1) Vitamin and mineral premix provided the following per kilogram of complete diet: vitamin A as retinyl acetate, 5,512 IU, vitamin D_3_ as cholecalciferol, 2,200 IU, vitamin E as _DL_-alpha-tocopheryl acetate, 30 IU, vitamin K_3_ as menadione nicotinamide bisulfite, 2.2 mg, vitamin B_12_, 27.6 μg, riboflavin, 4 mg, pantothenic acid as _DL_-calcium pantothenate, 14 mg, niacin, 30 mg, choline chloride, 400 mg, folacin, 0.7 mg, thiamin as thiamine mononitrate, 1.5 mg, pyridoxine as pyridoxine hydrochloride, 3 mg, biotin, 44 μg, Mn as MnO, 40 mg, Fe as FeSO_4_·H_2_O, 75 mg, Zn as ZnO, 75 mg, Cu as CuSO_4_·5H_2_O, 100 mg, I as KI, 0.3 mg, and Se as Na_2_SeO_3_, 0.3 mg.

FSBM, fermented soybean meal.

**Table 4:** Effect of experimental diets containing different fermented soybean meal (FSBM) sources on nutrient and energy digestibility and nitrogen balance in growing pigs

Items	Basal diet	FSBM diets No.									SEM	p-value

		1	2	3	4	5	6	7	8	9		
BW (kg)	32.1	37.0	34.9	36.8	36.3	36.9	34.4	34.5	35.3	34.8	1.4	0.263
DM intake (kg/d)	1.04	1.23	1.15	1.20	1.21	1.22	1.14	1.17	1.19	1.18	0.05	0.287
ATTD (%)												
DM	87.4^ab^	85.7^ab^	87.3^ab^	87.2^ab^	88.9^a^	88.0^ab^	85.8^ab^	86.6^ab^	84.4^b^	88.4^ab^	0.9	0.031
GE	87.2^ab^	86.0^ab^	87.7^ab^	87.4^ab^	89.3^a^	88.4^ab^	85.4^ab^	85.9^ab^	83.9^b^	88.5^a^	1.0	0.009
CP	75.6^c^	84.8^a^	86.6^a^	87.1^a^	89.2^a^	88.1^a^	85.0^ab^	85.5^a^	78.0^bc^	87.5^a^	1.4	<0.001
NDF	33.9^b^	39.2^ab^	43.1^ab^	54.2^a^	54.0^a^	45.0^a^	42.7^ab^	50.7^ab^	38.9^ab^	54.3^a^	3.8	<0.001
ADF	47.9^b^	56.7^ab^	65.8^a^	68.6^a^	66.5^a^	67.0^a^	57.7^ab^	59.3^ab^	50.3^b^	67.9^a^	3.0	<0.001
EE	53.8	54.0	57.2	59.9	66.5	57.7	52.8	56.0	56.0	62.6	3.6	0.182
OM	89.4^ab^	88.2^ab^	89.4^ab^	89.4^ab^	91.0^a^	90.2^ab^	88.0^ab^	88.7^ab^	86.5^b^	90.3^ab^	0.8	0.023
Nitrogen balance (g/d)												
Intake	15.20^b^	43.39^a^	40.90^a^	43.87^a^	43.35^a^	44.42^a^	39.31^a^	42.33^a^	42.79^a^	42.20^a^	1.79	<0.001
Fecal output	4.26^c^	7.39^b^	6.17^bc^	6.26^bc^	5.30^bc^	6.12^bc^	7.12^b^	6.84^bc^	10.57^a^	5.88^bc^	0.60	<0.001
Urinary output	6.72^b^	15.83^a^	14.26^a^	14.99^a^	14.12^a^	15.15^a^	13.46^ab^	12.23^ab^	15.35^a^	16.17^a^	1.55	0.004
Retention	4.22^b^	20.17^a^	20.47^a^	22.61^a^	23.93^a^	23.14^a^	18.73^a^	23.25^a^	16.87^a^	20.14^a^	1.81	<0.001

a–c Means in the same row with differing superscripts differ (p<0.05).

SEM, standard error of mean; BW, initial body weight; DM, dry matter; ATTD, apparent total tract digestibility; GE, gross energy; CP, crude protein; NDF, neutral dietary fiber; ADF, acid dietary fiber; EE, ether extract; OM, organic matter.

**Table 5:** Effect of experimental diets containing different fermented soybean meal (FSBM) sources on energy balance of growing pigs

Items	Basal diet	FSBM diets No.									SEM	p-value

		1	2	3	4	5	6	7	8	9		
Energy balance (kJ/kg BW^0.6^/d)												
ME intake	1,960	2,124	2,079	2,121	2,181	2,185	1,996	2,060	1,976	2,185	57	0.313
THP	1,282^c^	1,399^ab^	1,401^abc^	1,401^abc^	1,476^a^	1,391^abc^	1,429^ab^	1,403^abc^	1,349^bc^	1,416^ab^	27	<0.001
FHP	872	905	916	824	848	824	920	933	943	878	37	0.202
RE (kJ/kg BW^0.6^/d)												
RE_P_	77^b^	342^a^	363^a^	385^a^	414^a^	397^a^	334^a^	411^a^	296^a^	357^a^	28	<0.001
RE_L_	690	586	576	563	626	679	320	501	470	533	65	0.318
Total RE	768^a^	928^ab^	939^b^	948^ab^	1,040^b^	1,076^ab^	654^b^	912^b^	766^b^	891^ab^	58	<0.001
RQ												
Fed state	1.23	1.14	1.18	1.15	1.14	1.13	1.09	1.10	1.13	1.07	0.05	0.543
Fasted state	0.89	0.90	0.91	0.87	0.86	0.85	0.86	0.83	0.94	0.81	0.05	0.819
Energy utilization (%)												
UE-to-DE ratio	2.7	3.6	5. 6	3.5	4.5	3.1	4.0	4.1	4.3	4.6	0.7	0.184
ME-to-DE ratio	96.8	95.6	93.7	95.5	94.7	96.2	95.3	95.4	95.0	94.9	0. 7	0.113
NE-to-ME ratio	79.0^a^	77.2^ab^	76.7^ab^	73.9^bc^	71.9^c^	74.1^bc^	75.6^abc^	77.2^ab^	79.5^a^	75.3^abc^	1.0	<0.001
Energy values (MJ/kg DM)												
DE	15.7^bcd^	15.8^abcd^	16.3^abc^	16.2^abcd^	16.5^a^	16.2^abcd^	15.4^cde^	15. 5^cde^	14.9^e^	16.5^ab^	0.2	<0.001
ME	15.2^abc^	15.1^abc^	15.2^abc^	15. 5^abc^	15.6^a^	15.6^ab^	14. 7^cd^	14.7^bcd^	14.1^d^	15.6^a^	0.2	<0.001
NE	12.0^a^	11. 7^abc^	11. 7^abc^	11.5^abc^	11.3^bc^	11.5^abc^	11.2^c^	11.4^bc^	11.2^c^	11.8^ab^	0.1	<0.001

a–e Means in the same row with differing superscripts differ (p<0.05).

SEM, standard error of mean; BW, initial body weight; ME, metabolizable energy; THP, total heat production; FHP, fasting heat production; RE, retention energy; RE_P_, retention energy as protein; RE_L_, retention energy as lipids; RQ, respiratory quotient; UE, total urinary output of energy; DE, digestible energy; NE, net energy; DM, dry matter.

**Table 6:** The apparent total tract digestibility, energy utilization, and energy values of the fermented soybean meal (FSBM) samples

Items^1)^	FSBM No.									SEM	p-value

	1	2	3	4	5	6	7	8	9		
ATTD (%)											
GE	82.3^ab^	87.8^ab^	86.8^ab^	92.9^a^	90.0^ab^	82.4^ab^	84.0^ab^	77.2^b^	92.3^ab^	3.4	0.030
CP	88.9^a^	91.5^a^	92.0^a^	95.1^a^	93.4^a^	87.2^ab^	89.1^a^	78.8^b^	92.0^a^	1.9	<0.001
NDF	42.0	52.7	77.4	87.4	61.5	61.8	88.4	48.2	84.6	10.4	0.065
ADF	69.0^ab^	82.9^ab^	89.0^a^	88.8^a^	88.2^a^	68.0^ab^	75.1^ab^	53.9^b^	86.7^a^	6.5	0.003
OM	85.0^ab^	89.3^ab^	89.3^ab^	95.1^a^	92.4^ab^	84.4^ab^	86.7^ab^	79.1^b^	92.4^ab^	3.2	0.024
Energy utilization (%)											
ME-to-DE ratio	92.5	86.4	92.2	88.8	94.7	91.7	92.0	90.5	90.0	2.4	0.500
NE-to-ME ratio	87.5	81.6	70. 6	80.8	81.5	79.0	79.7	70.4	73.7	3.3	0.092
Energy values (MJ/kg DM)											
DE	16.52^bc^	17.72^ab^	17.83^ab^	18.44^a^	17.54^ab^	16.07^bc^	16.72^abc^	15.50^c^	18.40^a^	0.66	0.030
ME	15.28	15.30	16.44	16.37	16.69	14.62	15.26	13.98	16.72	0.67	0.066
NE	10.91	10.10	10.20	11.02	10.50	10.68	10.80	10.61	11.05	0.32	0.201

1) The ATTD and available energy of FSBM samples were calculated based on individual animal data using the difference method. As a result, each FSBM sample was represented by six replicate measurements for both ATTD and available energy, and these values were used for statistical analysis.

a–c Means in the same row with differing superscripts differ (p<0.05).

ATTD, apparent total tract digestibility; GE, gross energy; CP, crude protein; NDF, neutral dietary fiber; ADF, acid dietary fiber; OM, organic matter; ME, metabolizable energy; DE, digestible energy; NE, net energy; DM, dry matter.

**Table 7:** The apparent ileal digestibility (AID) and the averaged basal endogenous losses (BEL, g/kg dry matter intake) of crude protein and amino acids from different fermented soybean meal (FSBM) in Exp. 2

Items	The AID of FSBM diets No.									Mean	SEM	p-value	BEL^1)^

	1	2	3	4	5	6	7	8	9				
Crude protein	72.7	73.4	75.5	76.3	73.7	72.4	74.4	73.3	72.0	73.7	2.0	0.943	5.20
Indispensable amino acids													
Arginine	74.6	80.2	78.5	75.0	77.7	74.3	75.9	75.5	79.1	76.8	1.5	0.268	0.48
Histidine	70.7	80.1	74.2	69.9	72.2	74.2	75.2	71.8	77.2	74.0	2.1	0.063	0.20
Isoleucine	76.4	80.1	80.5	78.2	76.6	80.0	77.8	78.9	83.3	79.1	1.4	0.115	0.19
Leucine	75.6	78.2	77.3	74.9	77.9	74.2	74.7	73.2	82.6	76.5	1.6	0.310	0.79
Lysine	57.7^b^	78.5^a^	73.4^ab^	75.3^a^	71.0^ab^	69.7^ab^	73.3^ab^	68.0^ab^	78.4^a^	76.0	1.9	0.036	0.28
Methionine	53.7^b^	68.2^ab^	64.3^ab^	59.2^ab^	69.7^a^	58.9^ab^	70.7^a^	59.5^ab^	69.5^ab^	63.7	1.7	0.005	0.30
Phenylalanine	69.5	75.7	72.8	72.0	72.4	67.2	71.9	71.7	79.1	72.4	1.5	0.104	0.23
Threonine	75.8	81.1	79.1	77.7	76.6	77.2	76.2	77.3	83.4	78.3	2.2	0.136	0.19
Tryptophan	76.8^b^	84.0^ab^	83.6^ab^	82.5^ab^	80.8^ab^	76.9^b^	82.5^ab^	81. 7^ab^	86.9^a^	81.7	2.1	0.043	0.16
Valine	79.2	82.4	81.8	80.6	78.4	78.1	82.0	81.5	85.7	81.1	1.6	0.171	0.26
Dispensable amino acids													
Alanine	77.7	83.3	80.3	78.4	76.9	74.0	79.1	80.7	80.7	79.0	1.9	0.182	0.11
Asparagine	78.3^bc^	83.9^abc^	85.4^ab^	82.9^abc^	81.2^abc^	78.3^c^	81.4^abc^	82.9^abc^	86.1^a^	82.3	1.8	0.008	0.17
Cystine	75.3	85.2	82.4	80.7	81.0	77.5	81.2	81.0	83.5	80.9	2.0	0.105	0.09
Glutamic acid	70.8	75.2	76.4	72.6	74.2	70.0	71.6	74.0	76.3	73. 5	2.3	0.315	0.27
Glycine	87.2	90.2	88.8	87.8	87.2	87.2	89.1	88.8	90.5	88.5	2.5	0.142	0.14
Proline	74.8	78.3	72.6	72.2	70.7	73.8	74.6	73.0	76.1	74.0	2.4	0.496	0.08
Serine	76.4	77.7	76.0	76.8	75.7	72.9	77.7	76.6	82.6	76.9	1.7	0.217	0.05
Tyrosine	72.0^ab^	74.8^ab^	72.0^ab^	75.3^ab^	77.0^ab^	70.8^b^	71.6^ab^	74.4^ab^	84.4^a^	74.7	1.9	0.025	0.05

1) The BEL of FSBM presented in this table represents the average value derived from nitrogen-free diet.

a–c Means in the same row with differing superscripts differ (p<0.05).

SEM, standard error of mean.

**Table 8:** The standardized ileal digestibility values of crude protein and amino acids from different fermented soybean meal (FSBM) in Exp. 2

Items	FSBM diets No.									Mean	SEM	p-value

	1	2	3	4	5	6	7	8	9			
Crude protein	72.8	74.8	78.0	76.4	76.0	72.5	75.0	75. 5	75.8	75.2	2.0	0.653
Indispensable amino acids												
Arginine	87.2	90.3	88.5	87. 9	87. 7	85.3	90.3	86.2	91.8	88.3	1.5	0.108
Histidine	75.4^c^	85.2^a^	85.0^a^	80.8^abc^	81.5^abc^	77.6^bc^	82.9^ab^	81.2^abc^	84.3^a^	81.5	2.1	0.043
Isoleucine	79.3^b^	82.5^a^	83.5^a^	80.7^ab^	79.4^ab^	75.9^b^	83.5^ab^	81.6^ab^	86.5^a^	81.4	1.4	<0.001
Leucine	76.9^ab^	84.0^ab^	84.9^a^	82.5^ab^	82.1^ab^	77.0^b^	83.6^ab^	81.8^ab^	87.7^a^	82.3	1.6	0.006
Lysine	70.9	75.3	77.0	72.8	75.5	72.1	76.8	74.1	77.7	74.7	1.9	0.261
Methionine	76.5^ab^	77.8^ab^	76.0^b^	76.9^b^	76.4^b^	73.0^b^	79.6^ab^	76.7^b^	84.6^a^	77.5	1.7	0.004
Phenylalanine	78.3^b^	83.9^ab^	85.2^ab^	83.0^ab^	81.9^ab^	78.4^b^	85.4^ab^	83.0^ab^	87.0^a^	82.9	1.5	0.003
Threonine	70.9^ab^	80.2^a^	76.4^ab^	70.0^b^	73.5^ab^	76.9^ab^	75.6^ab^	71.9^ab^	78.2^ab^	74.8	2.2	0.022
Tryptophan	72.1^b^	74.9^ab^	73.6^ab^	76.8^ab^	77.8^ab^	70.9^b^	75.5^ab^	74.5^ab^	83.9^a^	75.5	2.1	0.018
Valine	75.9^b^	81.2^ab^	81.2^ab^	77.8^ab^	77.5^ab^	77.3^ab^	80.8^ab^	77.4^ab^	84.3^a^	79.3	1.6	0.021
Dispensable amino acids												
Alanine	72.5^ab^	78.0^a^	75.6^ab^	73.2^ab^	74.0^ab^	67.3^b^	75.9^ab^	71.9^ab^	80.3^a^	74.3	1.9	0.007
Asparagine	74.7^bc^	80.3^a^	80.6^a^	75.1^bc^	79.1^abc^	74.4^c^	80.9^a^	75.6^bc^	80.2^ab^	77.9	1.8	0.033
Cystine	74.9^ab^	78.4^ab^	71.4^b^	72.3^ab^	71.2^b^	73.9^ab^	81.2^a^	73.1^ab^	76.6^ab^	74.8	2.0	0.019
Glutamic acid	75.7	78.3	80.0	75.0	79.4	74.3	80.9	73.3	83.5	77.8	2.3	0.052
Glycine	53.9^c^	70.2^ab^	69.5^ab^	62.8^abc^	73.7^a^	61.1^bc^	69.8^ab^	61.4^bc^	69.6^ab^	65.8	2.5	0.006
Proline	57.9^b^	78.6^a^	78.6^a^	75.5^a^	70.8^a^	74.3^a^	78.5^a^	71.0^a^	77.3^a^	73.6	2.4	0.003
Serine	76.5	80.1	81.9	78.3	79.4	77.7	82.8	79.0	84.0	80.0	1.7	0.093
Tyrosine	77.8	83.3	82.1	78.5	79.5	74.1	81.1	80.8	82.1	79.9	1.9	0.064

a–c Means in the same row with differing superscripts differ (p<0.05).

SEM, standard error of mean.

**Table 9:** Correlation coefficients between chemical composition, energy values, and standardized ileal digestibility of the nine fermented soybean meal samples

Items	DM	CP	EE	CF	NDF	ADF	Ash	GE	NE	SID_CP_
CP	−0.23									
EE	−0.27	−0.02								
CF	0.36	−0.56	0.21							
NDF	0.12	0.18	0.51	0.49						
ADF	0.17	0.53	0.25	0.02	0.81^**^					
Ash	0.57	−0.01	−0.60	0.03	−0.25	−0.05				
GE	0.75^*^	0.18	0.17	0.22	0.42	0.41	0.14			
NE	0.78^*^	0.17	−0.38	0.09	−0.12	−0.01	0.47	0.76^*^		
SID_CP_	−0.48	0.61	0.27	−0.33	0.27	0.54	−0.17	−0.23	−0.39	
SID_Lys_	−0.27	0.19	0.51	0.18	0.69^*^	0.59	−0.70^*^	0.11	−0.34	0.54

* p<0.05;

** p<0.01.

DM, dry matter; CP, crude protein; EE, ether extract; CF, crude fiber; NDF, neutral dietary fiber; ADF, acid dietary fiber; GE, gross energy; NE, net energy; SID_CP_, the standardized ileal digestibility of crude protein; SID_Lys_, the standardized ileal digestibility of Lys.

**Table 10:** Stepwise regression equations to estimate net energy (MJ/kg DM), standardized ileal digestibility of crude protein (SID_CP_) and lysine (SID_Lys_, %) from nutrient concentrates of fermented soybean meal (DM basis)

Number	Equations for NE, SID_CP_, and SID_Lys_	Statistics			

		R^2^	RMSE	AIC	p-value
1	NE = −13.31–0.47×EE–0.066×NDF+1.32×GE	0.92	0.16	16.84	0.005
2	NE = −10.94–0.66×EE+1.16×GE	0.85	0.19	10.30	0.008
3	NE = −13.94–0.11×NDF+1.35×GE	0.81	0.22	12.29	<0.001
4	SID_CP_ = 118.24–0.58×DM+1.83×ADF	0.63	1.21	43.55	0.052
5	SID_CP_ = 117.42–0.58×DM–0.40×NDF+2.99×ADF	0.71	1.16	52.98	0.079
6	SID_Lys_ = 202.16+6.13×ADF–24.38×Ash	0.79	3.48	62.33	0.003
7	SID_Lys_ = 251.57–1.10×CP+7.08×ADF–24.30×Ash	0.81	3.63	73.47	0.032
8	SID_Lys_ = 196.63+1.56×CF+6.08×ADF–24.56×Ash	0.83	3.47	72.66	0.022

DM, dry matter; SID_CP_, the standardized ileal digestibility of crude protein; SID_Lys_, the standardized ileal digestibility of Lys; NE, net energy; R^2^, the coefficient of determination; RMSE, root mean square error; AIC, Akaike’s information criterion; EE, ether extract; NDF, neutral dietary fiber; GE, gross energy; CP, crude protein; ADF, acid dietary fiber; Lys, lysine; CF, crude fiber.
